# Diethyl 7,8-dibromo-4,11-dioxo-11b,11c-dihydro-5*H*,10*H*-2-oxa-3a,4a,10a,11a-tetra­azabenz[*f*]indeno[2,1,7,7a-*ija*]azulene-11b,11c-dicarboxyl­ate

**DOI:** 10.1107/S160053680903284X

**Published:** 2009-08-26

**Authors:** Yan Chen, Nengfang She

**Affiliations:** a Key Laboratory of Pesticides and Chemical Biology of the Ministry of Education, College of Chemistry, Central China Normal University, Wuhan 430079, People’s Republic of China

## Abstract

The title compound, C_20_H_20_Br_2_N_4_O_7_, is an inter­mediate for mol­ecular clips. The seven- and six-membered rings have chair conformations, while the five-membered rings adopt envelope conformations. In the crystal structure, weak C—H⋯O and C—H⋯Br inter­actions link the mol­ecules into a three-dimensional network. The eth­oxy and ethyl groups are disordered over two orientations, with occupancy ratios of 0.735 (16):0.265 (16) and 0.51 (2):0.49 (2), respectively.

## Related literature

For general background, see: Burnett *et al.* (2003 ▶). For a related structure, see: Wu *et al.* (2002 ▶). For ring-puckering parameters, see: Cremer & Pople (1975 ▶). For bond-length data, see: Allen *et al.* (1987 ▶).
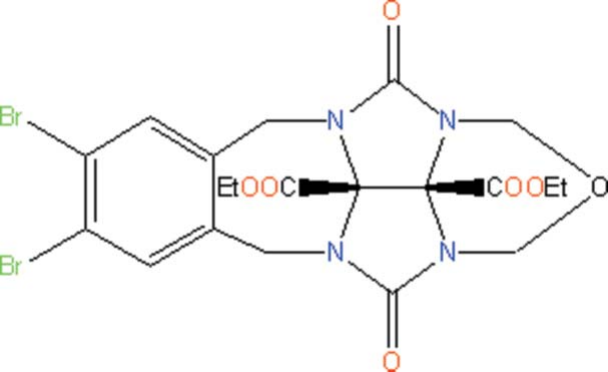


## Experimental

### 

#### Crystal data

C_20_H_20_Br_2_N_4_O_7_
*M* *_r_* = 588.22
Monoclinic, 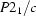

*a* = 12.4679 (10) Å
*b* = 15.1505 (13) Å
*c* = 11.5383 (10) Å
β = 90.189 (1)°
*V* = 2179.5 (3) Å^3^
*Z* = 4
Mo *K*α radiation
μ = 3.77 mm^−1^
*T* = 292 K
0.30 × 0.20 × 0.20 mm

#### Data collection

Bruker SMART 4K CCD area-detector diffractometer
Absorption correction: none
18344 measured reflections
4736 independent reflections
2983 reflections with *I* > 2σ(*I*)
*R*_int_ = 0.097

#### Refinement

*R*[*F* ^2^ > 2σ(*F* ^2^)] = 0.050
*wR*(*F* ^2^) = 0.134
*S* = 0.91
4736 reflections
349 parameters
30 restraints
H-atom parameters constrained
Δρ_max_ = 1.00 e Å^−3^
Δρ_min_ = −0.59 e Å^−3^

### 

Data collection: *SMART* (Bruker, 2001 ▶); cell refinement: *SAINT* (Bruker, 2001 ▶); data reduction: *SAINT*; program(s) used to solve structure: *SHELXS97* (Sheldrick, 2008 ▶); program(s) used to refine structure: *SHELXL97* (Sheldrick, 2008 ▶); molecular graphics: *PLATON* (Spek, 2009 ▶); software used to prepare material for publication: *SHELXTL* (Sheldrick, 2008 ▶).

## Supplementary Material

Crystal structure: contains datablocks I, global. DOI: 10.1107/S160053680903284X/hk2753sup1.cif

Structure factors: contains datablocks I. DOI: 10.1107/S160053680903284X/hk2753Isup2.hkl

Additional supplementary materials:  crystallographic information; 3D view; checkCIF report

## Figures and Tables

**Table 1:** Hydrogen-bond geometry (Å, °)

*D*—H⋯*A*	*D*—H	H⋯*A*	*D*⋯*A*	*D*—H⋯*A*
C19—H19*B*⋯O2^i^	0.97	2.51	3.353 (4)	146
C17—H17*A*⋯Br1^ii^	0.97	2.94	3.625 (10)	129
C8—H8*B*⋯O2^iii^	0.97	2.39	3.324 (4)	161

Symmetry codes: (i) 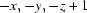; (ii) 
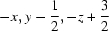
; (iii) 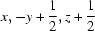.

**Fig. 1.**
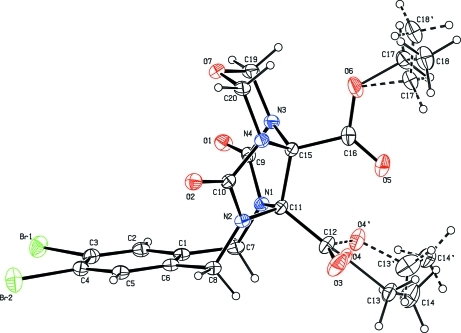
The molecular structure of the title molecule, with the atom-numbering scheme. Displacement ellipsoids are drawn at the 50% probability level.

**Fig. 2.**
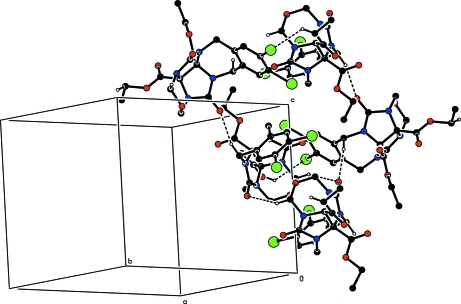
A partial packing diagram. Hydrogen bonds are shown as dashed lines. H atoms not involved in hydrogen bonding have been omitted for clarity.

## supplementary crystallographic information

### Comment

Diethoxycarbonyl glycoluril bearing a range of electron withdrawing functional groups on its convex face is an important building block for both molecular and supramolecular chemistry (Burnett *et al.*, 2003). The title compound derived from diethoxycarbonyl glycoluril is an important intermediate for methylene-bridged glycoluril dimers, and we report herein its crystal structure.

In the molecule of the title compound, (Fig. 1), the bond lengths (Allen *et al.*, 1987) and angles are within normal ranges. Ring A (C1-C6) is, of course, planar. The seven-membered ring B (N1/N2/C1/C6-C8/c11) is not planar, having total puckering amplitude, Q_T_, of 2.878 (2) Å (Cremer & Pople, 1975), and resembles chair conformation. Rings C (N1/N3/C9/C11/C15) and D (N2/N4/C10/C11/C15) adopt envelope conformations with atoms N1 and N4 displaced by -0.208 (3) and -0.174 (3) Å from the planes of the other ring atoms, respectively, while ring E (O7/N3/N4/C15/C19/C20) is not planar, having total puckering amplitude, Q_T_, of 0.429 (2) Å and adopts chair conformation [φ = -90.18 (3) and θ = 91.16 (3) °] (Cremer & Pople, 1975).

In the crystal structure, weak C-H···O and C-H···Br interactions link the molecules into a three-dimensional network (Fig. 2), in which they may be effective in the stabilization of the structure.

### Experimental

The title compound was synthesized according to a literature method (Wu *et al.*, 2002). Crystals suitable for X-ray analysis were obtained by slow evaporation of a dichloride methane solution at 283 K.

### Refinement

H atoms were positioned geometrically, with C-H = 0.93, 0.97 and 0.96 Å for aromatic, methylene and methyl H, respectively, and constrained to ride on their parent atoms, with U_iso_(H) = 1.2U_eq_(C). The ethoxy and ethyl groups attached at C12 and O6, respectively, are disordered over two orientations. During the refinement process, the disordered O4, C13, C14, H13A, H13B, H14A, H14B, H14C and O4', C13', C14', H13C, H13D, H14D, H14E, H14F atoms were refined with occupancies of 0.735 (16) and 0.265 (16), while C17, C18, H17A, H17B, H18A, H18B, H18C and C17', C18', H17C, H17D, H18D, H18E, H18F atoms were refined with occupancies of 0.51 (2) and 0.49 (2), respectively, by applying some restraints.

### Crystal data

**Table:**

C_20_H_20_Br_2_N_4_O_7_	*F*(000) = 1176
*M**_r_* = 588.22	*D*_x_ = 1.793 Mg m^−^^3^
Monoclinic, *P*2_1_/*c*	Mo *K*α radiation, λ = 0.71073 Å
Hall symbol: -P 2ybc	Cell parameters from 5213 reflections
*a* = 12.4679 (10) Å	θ = 2.2–26.1°
*b* = 15.1505 (13) Å	µ = 3.77 mm^−^^1^
*c* = 11.5383 (10) Å	*T* = 292 K
β = 90.189 (1)°	Block, colorless
*V* = 2179.5 (3) Å^3^	0.30 × 0.20 × 0.20 mm
*Z* = 4	

### Data collection

**Table:**

Bruker SMART 4K CCD area-detector diffractometer	2983 reflections with *I* > 2σ(*I*)
Radiation source: fine-focus sealed tube	*R*_int_ = 0.097
graphite	θ_max_ = 27.0°, θ_min_ = 2.1°
φ and ω scans	*h* = −15→15
18344 measured reflections	*k* = −19→19
4736 independent reflections	*l* = −14→13

### Refinement

**Table:**

Refinement on *F*^2^	Primary atom site location: structure-invariant direct methods
Least-squares matrix: full	Secondary atom site location: difference Fourier map
*R*[*F*^2^ > 2σ(*F*^2^)] = 0.050	Hydrogen site location: inferred from neighbouring sites
*wR*(*F*^2^) = 0.134	H-atom parameters constrained
*S* = 0.91	*w* = 1/[σ^2^(*F*_o_^2^) + (0.08*P*)^2^] where *P* = (*F*_o_^2^ + 2*F*_c_^2^)/3
4736 reflections	(Δ/σ)_max_ < 0.001
349 parameters	Δρ_max_ = 1.00 e Å^−^^3^
30 restraints	Δρ_min_ = −0.58 e Å^−^^3^

### Special details

**Table:**

Geometry. All e.s.d.'s (except the e.s.d. in the dihedral angle between two l.s. planes) are estimated using the full covariance matrix. The cell e.s.d.'s are taken into account individually in the estimation of e.s.d.'s in distances, angles and torsion angles; correlations between e.s.d.'s in cell parameters are only used when they are defined by crystal symmetry. An approximate (isotropic) treatment of cell e.s.d.'s is used for estimating e.s.d.'s involving l.s. planes.
Refinement. Refinement of *F*^2^ against ALL reflections. The weighted *R*-factor *wR* and goodness of fit *S* are based on *F*^2^, conventional *R*-factors *R* are based on *F*, with *F* set to zero for negative *F*^2^. The threshold expression of *F*^2^ > σ(*F*^2^) is used only for calculating *R*-factors(gt) *etc*. and is not relevant to the choice of reflections for refinement. *R*-factors based on *F*^2^ are statistically about twice as large as those based on *F*, and *R*- factors based on ALL data will be even larger.

### Fractional atomic coordinates and isotropic or equivalent isotropic displacement parameters (Å^2^)

**Table:**

	*x*	*y*	*z*	*U*_iso_*/*U*_eq_	Occ. (<1)
Br1	−0.39306 (3)	0.14276 (3)	0.75601 (4)	0.06508 (19)	
Br2	−0.31776 (4)	0.30716 (4)	0.57266 (4)	0.0716 (2)	
O1	−0.01869 (19)	−0.05766 (16)	0.7742 (2)	0.0470 (7)	
O2	0.1247 (2)	0.18343 (17)	0.4670 (2)	0.0461 (6)	
O3	0.3227 (3)	0.2018 (2)	0.8002 (3)	0.0737 (10)	
O4	0.2543 (5)	0.1138 (5)	0.9343 (4)	0.0491 (16)	0.735 (16)
O4'	0.2838 (13)	0.0817 (11)	0.9206 (11)	0.053 (4)	0.265 (16)
O5	0.3918 (3)	0.0190 (3)	0.7638 (4)	0.1048 (15)	
O6	0.3624 (2)	−0.0744 (3)	0.6229 (3)	0.0823 (11)	
O7	0.10231 (19)	−0.05214 (17)	0.4804 (2)	0.0465 (6)	
N1	0.0940 (2)	0.05941 (18)	0.8063 (2)	0.0342 (6)	
N2	0.1467 (2)	0.16772 (18)	0.6633 (2)	0.0344 (6)	
N3	0.1438 (2)	−0.04468 (18)	0.6808 (3)	0.0375 (7)	
N4	0.2148 (2)	0.06373 (19)	0.5477 (2)	0.0362 (7)	
C1	−0.0639 (3)	0.1581 (2)	0.7972 (3)	0.0368 (8)	
C2	−0.1724 (3)	0.1369 (2)	0.8074 (3)	0.0429 (9)	
H2	−0.1937	0.0938	0.8599	0.052*	
C3	−0.2483 (3)	0.1793 (3)	0.7405 (3)	0.0434 (9)	
C4	−0.2182 (3)	0.2441 (3)	0.6642 (3)	0.0453 (9)	
C5	−0.1106 (3)	0.2646 (2)	0.6516 (3)	0.0407 (8)	
H5	−0.0903	0.3077	0.5988	0.049*	
C6	−0.0329 (3)	0.2218 (2)	0.7163 (3)	0.0368 (8)	
C7	0.0170 (3)	0.1112 (3)	0.8718 (3)	0.0423 (9)	
H7A	0.0555	0.1545	0.9177	0.051*	
H7B	−0.0206	0.0725	0.9249	0.051*	
C8	0.0838 (3)	0.2443 (2)	0.6985 (3)	0.0381 (8)	
H8A	0.0895	0.2897	0.6396	0.046*	
H8B	0.1131	0.2679	0.7700	0.046*	
C9	0.0625 (3)	−0.0180 (2)	0.7541 (3)	0.0358 (8)	
C10	0.1571 (3)	0.1427 (2)	0.5517 (3)	0.0345 (8)	
C11	0.1813 (3)	0.1016 (2)	0.7450 (3)	0.0352 (8)	
C12	0.2663 (3)	0.1435 (3)	0.8275 (3)	0.0487 (10)	
C13	0.3325 (7)	0.1493 (6)	1.0189 (6)	0.070 (3)	0.735 (16)
H13A	0.3212	0.2121	1.0303	0.084*	0.735 (16)
H13B	0.4052	0.1402	0.9917	0.084*	0.735 (16)
C13'	0.3735 (18)	0.0999 (19)	1.0039 (17)	0.081 (7)	0.265 (16)
H13C	0.3752	0.1621	1.0236	0.097*	0.265 (16)
H13D	0.4417	0.0841	0.9695	0.097*	0.265 (16)
C14	0.3146 (8)	0.1006 (8)	1.1289 (7)	0.106 (4)	0.735 (16)
H14A	0.2406	0.1055	1.1507	0.128*	0.735 (16)
H14B	0.3590	0.1253	1.1888	0.128*	0.735 (16)
H14C	0.3327	0.0395	1.1182	0.128*	0.735 (16)
C14'	0.354 (3)	0.045 (3)	1.112 (2)	0.165 (17)	0.265 (16)
H14D	0.2780	0.0423	1.1272	0.198*	0.265 (16)
H14E	0.3899	0.0710	1.1769	0.198*	0.265 (16)
H14F	0.3804	−0.0140	1.1001	0.198*	0.265 (16)
C15	0.2217 (2)	0.0256 (2)	0.6629 (3)	0.0344 (8)	
C16	0.3359 (3)	−0.0087 (3)	0.6886 (4)	0.0492 (10)	
C17	0.4520 (7)	−0.1364 (8)	0.6416 (14)	0.050 (3)	0.51 (2)
H17A	0.4337	−0.1946	0.6128	0.060*	0.51 (2)
H17B	0.4693	−0.1408	0.7234	0.060*	0.51 (2)
C17'	0.4760 (10)	−0.0973 (16)	0.6529 (15)	0.082 (6)	0.49 (2)
H17C	0.4787	−0.1386	0.7171	0.098*	0.49 (2)
H17D	0.5164	−0.0449	0.6735	0.098*	0.49 (2)
C18	0.5452 (7)	−0.0986 (11)	0.5752 (14)	0.066 (4)	0.51 (2)
H18A	0.5265	−0.0941	0.4947	0.079*	0.51 (2)
H18B	0.6064	−0.1366	0.5838	0.079*	0.51 (2)
H18C	0.5622	−0.0410	0.6049	0.079*	0.51 (2)
C18'	0.5196 (12)	−0.1389 (17)	0.5435 (14)	0.096 (6)	0.49 (2)
H18D	0.4778	−0.1901	0.5242	0.115*	0.49 (2)
H18E	0.5930	−0.1559	0.5558	0.115*	0.49 (2)
H18F	0.5158	−0.0971	0.4811	0.115*	0.49 (2)
C19	0.1211 (3)	−0.1018 (2)	0.5829 (3)	0.0459 (9)	
H19A	0.1812	−0.1414	0.5707	0.055*	
H19B	0.0584	−0.1375	0.5997	0.055*	
C20	0.1918 (3)	0.0019 (3)	0.4536 (3)	0.0435 (9)	
H20A	0.1773	0.0346	0.3830	0.052*	
H20B	0.2541	−0.0350	0.4403	0.052*	

### Atomic displacement parameters (Å^2^)

**Table:**

	*U*^11^	*U*^22^	*U*^33^	*U*^12^	*U*^13^	*U*^23^
Br1	0.0421 (2)	0.0827 (4)	0.0705 (3)	−0.0127 (2)	0.0010 (2)	−0.0028 (2)
Br2	0.0526 (3)	0.0970 (4)	0.0652 (3)	0.0130 (2)	−0.0028 (2)	0.0176 (3)
O1	0.0421 (14)	0.0383 (14)	0.0606 (18)	−0.0069 (11)	0.0073 (12)	0.0001 (12)
O2	0.0572 (15)	0.0466 (15)	0.0344 (15)	0.0013 (12)	−0.0013 (12)	0.0038 (12)
O3	0.070 (2)	0.074 (2)	0.077 (2)	−0.0353 (17)	−0.0222 (17)	0.0031 (18)
O4	0.045 (3)	0.067 (4)	0.035 (3)	0.001 (2)	−0.016 (2)	−0.003 (2)
O4'	0.031 (6)	0.088 (9)	0.039 (6)	0.006 (6)	−0.004 (5)	−0.006 (6)
O5	0.058 (2)	0.129 (4)	0.127 (3)	0.027 (2)	−0.050 (2)	−0.051 (3)
O6	0.0556 (18)	0.107 (3)	0.084 (2)	0.0454 (18)	−0.0168 (17)	−0.028 (2)
O7	0.0470 (14)	0.0474 (15)	0.0451 (16)	0.0019 (12)	−0.0088 (12)	−0.0125 (12)
N1	0.0360 (14)	0.0362 (15)	0.0303 (15)	−0.0013 (12)	0.0016 (12)	−0.0024 (13)
N2	0.0391 (15)	0.0336 (15)	0.0305 (16)	−0.0023 (12)	0.0009 (12)	−0.0027 (12)
N3	0.0387 (15)	0.0336 (16)	0.0401 (17)	−0.0002 (12)	−0.0002 (13)	−0.0040 (13)
N4	0.0375 (14)	0.0399 (16)	0.0311 (16)	0.0030 (12)	−0.0010 (12)	−0.0026 (13)
C1	0.0437 (19)	0.0347 (19)	0.0320 (19)	0.0005 (15)	0.0056 (15)	−0.0098 (15)
C2	0.049 (2)	0.042 (2)	0.038 (2)	−0.0012 (17)	0.0076 (17)	−0.0068 (17)
C3	0.0372 (18)	0.047 (2)	0.046 (2)	−0.0059 (16)	0.0066 (16)	−0.0110 (18)
C4	0.0426 (19)	0.050 (2)	0.043 (2)	0.0057 (17)	−0.0009 (16)	−0.0114 (18)
C5	0.049 (2)	0.0367 (19)	0.037 (2)	0.0009 (16)	0.0057 (16)	−0.0064 (16)
C6	0.0452 (19)	0.0327 (18)	0.033 (2)	−0.0001 (15)	0.0026 (15)	−0.0093 (15)
C7	0.050 (2)	0.046 (2)	0.032 (2)	−0.0033 (17)	0.0086 (16)	−0.0054 (17)
C8	0.0440 (18)	0.0330 (18)	0.037 (2)	−0.0029 (15)	0.0020 (16)	−0.0044 (15)
C9	0.0354 (17)	0.0367 (19)	0.035 (2)	0.0035 (15)	−0.0008 (14)	0.0037 (15)
C10	0.0327 (16)	0.0355 (19)	0.035 (2)	−0.0065 (14)	−0.0005 (14)	−0.0015 (16)
C11	0.0332 (16)	0.0411 (19)	0.0311 (19)	−0.0037 (14)	−0.0038 (14)	−0.0035 (15)
C12	0.044 (2)	0.060 (3)	0.042 (2)	−0.0079 (19)	−0.0106 (18)	−0.006 (2)
C13	0.071 (4)	0.069 (5)	0.070 (5)	−0.005 (4)	−0.039 (4)	−0.001 (4)
C13'	0.092 (11)	0.080 (11)	0.071 (10)	−0.014 (8)	−0.004 (8)	−0.021 (8)
C14	0.119 (7)	0.133 (9)	0.066 (6)	0.014 (7)	−0.041 (5)	0.008 (5)
C14'	0.166 (19)	0.165 (19)	0.165 (19)	0.001 (10)	−0.012 (10)	−0.006 (10)
C15	0.0315 (16)	0.0384 (19)	0.0334 (19)	0.0004 (14)	−0.0028 (14)	−0.0037 (15)
C16	0.0362 (19)	0.060 (3)	0.051 (3)	0.0025 (18)	−0.0016 (18)	0.007 (2)
C17	0.045 (5)	0.046 (6)	0.059 (8)	0.004 (4)	−0.002 (4)	−0.004 (5)
C17'	0.049 (8)	0.081 (12)	0.115 (11)	0.036 (7)	−0.037 (8)	−0.039 (10)
C18	0.043 (5)	0.089 (10)	0.065 (9)	0.014 (5)	0.009 (5)	0.011 (7)
C18'	0.057 (8)	0.104 (14)	0.127 (15)	0.035 (8)	−0.016 (8)	−0.013 (10)
C19	0.045 (2)	0.037 (2)	0.056 (3)	−0.0023 (16)	0.0008 (18)	−0.0112 (19)
C20	0.048 (2)	0.048 (2)	0.034 (2)	0.0065 (17)	0.0040 (16)	−0.0097 (17)

### Geometric parameters (Å, °)

**Table:**

Br1—C3	1.896 (3)	C13'—C14'	1.522 (10)
Br2—C4	1.887 (4)	C13'—H13C	0.9700
O4—C13	1.479 (6)	C13'—H13D	0.9700
O4'—C13'	1.498 (10)	C14—H14A	0.9600
C1—C2	1.396 (5)	C14—H14B	0.9600
C1—C6	1.399 (5)	C14—H14C	0.9600
C1—C7	1.503 (5)	C14'—H14D	0.9600
C2—C3	1.378 (5)	C14'—H14E	0.9600
C2—H2	0.9300	C14'—H14F	0.9600
C3—C4	1.372 (6)	C15—N4	1.451 (4)
C4—C5	1.386 (5)	C15—N3	1.457 (4)
C5—C6	1.382 (5)	C15—C16	1.544 (5)
C5—H5	0.9300	C16—O5	1.188 (5)
C6—C8	1.509 (5)	C16—O6	1.294 (5)
C7—N1	1.453 (4)	C17—O6	1.475 (8)
C7—H7A	0.9700	C17—C18	1.507 (9)
C7—H7B	0.9700	C17—H17A	0.9700
C8—N2	1.459 (4)	C17—H17B	0.9700
C8—H8A	0.9700	C17'—O6	1.497 (8)
C8—H8B	0.9700	C17'—C18'	1.514 (10)
C9—O1	1.199 (4)	C17'—H17C	0.9700
C9—N1	1.376 (4)	C17'—H17D	0.9700
C9—N3	1.383 (4)	C18—H18A	0.9600
C10—O2	1.223 (4)	C18—H18B	0.9600
C10—N2	1.349 (4)	C18—H18C	0.9600
C10—N4	1.396 (4)	C18'—H18D	0.9600
C11—N2	1.440 (4)	C18'—H18E	0.9600
C11—N1	1.450 (4)	C18'—H18F	0.9600
C11—C12	1.558 (5)	C19—O7	1.421 (5)
C11—C15	1.575 (5)	C19—N3	1.451 (5)
C12—O3	1.173 (5)	C19—H19A	0.9700
C12—O4	1.321 (6)	C19—H19B	0.9700
C12—O4'	1.441 (10)	C20—O7	1.419 (4)
C13—C14	1.485 (8)	C20—N4	1.462 (5)
C13—H13A	0.9700	C20—H20A	0.9700
C13—H13B	0.9700	C20—H20B	0.9700
			
C12—O4—C13	114.5 (5)	O3—C12—C11	123.3 (4)
C12—O4'—C13'	118.0 (14)	O4—C12—C11	110.7 (4)
C16—O6—C17	126.8 (7)	O4'—C12—C11	107.0 (6)
C16—O6—C17'	106.6 (6)	O4—C13—C14	106.4 (5)
C20—O7—C19	111.1 (3)	O4—C13—H13A	110.4
C9—N1—C11	112.1 (3)	C14—C13—H13A	110.4
C9—N1—C7	120.0 (3)	O4—C13—H13B	110.4
C11—N1—C7	120.9 (3)	C14—C13—H13B	110.4
C10—N2—C11	113.5 (3)	H13A—C13—H13B	108.6
C10—N2—C8	122.9 (3)	O4'—C13'—C14'	107.5 (13)
C11—N2—C8	122.1 (3)	O4'—C13'—H13C	110.2
C9—N3—C19	120.6 (3)	C14'—C13'—H13C	110.2
C9—N3—C15	111.4 (3)	O4'—C13'—H13D	110.2
C19—N3—C15	117.0 (3)	C14'—C13'—H13D	110.2
C10—N4—C15	109.9 (3)	H13C—C13'—H13D	108.5
C10—N4—C20	118.3 (3)	C13'—C14'—H14D	109.5
C15—N4—C20	115.9 (3)	C13'—C14'—H14E	109.5
C2—C1—C6	119.0 (3)	H14D—C14'—H14E	109.5
C2—C1—C7	119.5 (3)	C13'—C14'—H14F	109.5
C6—C1—C7	121.5 (3)	H14D—C14'—H14F	109.5
C3—C2—C1	120.6 (4)	H14E—C14'—H14F	109.5
C3—C2—H2	119.7	N4—C15—N3	112.6 (3)
C1—C2—H2	119.7	N4—C15—C16	111.2 (3)
C4—C3—C2	120.3 (3)	N3—C15—C16	109.9 (3)
C4—C3—Br1	122.1 (3)	N4—C15—C11	104.0 (3)
C2—C3—Br1	117.6 (3)	N3—C15—C11	103.6 (2)
C3—C4—C5	119.7 (4)	C16—C15—C11	115.3 (3)
C3—C4—Br2	122.7 (3)	O5—C16—O6	123.3 (4)
C5—C4—Br2	117.6 (3)	O5—C16—C15	124.0 (4)
C6—C5—C4	121.0 (4)	O6—C16—C15	112.6 (3)
C6—C5—H5	119.5	O6—C17—C18	105.6 (7)
C4—C5—H5	119.5	O6—C17—H17A	110.6
C5—C6—C1	119.3 (3)	C18—C17—H17A	110.6
C5—C6—C8	119.7 (3)	O6—C17—H17B	110.6
C1—C6—C8	121.0 (3)	C18—C17—H17B	110.6
N1—C7—C1	113.6 (3)	H17A—C17—H17B	108.8
N1—C7—H7A	108.9	O6—C17'—C18'	104.3 (7)
C1—C7—H7A	108.9	O6—C17'—H17C	110.9
N1—C7—H7B	108.9	C18'—C17'—H17C	110.9
C1—C7—H7B	108.9	O6—C17'—H17D	110.9
H7A—C7—H7B	107.7	C18'—C17'—H17D	110.9
N2—C8—C6	112.2 (3)	H17C—C17'—H17D	108.9
N2—C8—H8A	109.2	C17'—C18'—H18D	109.5
C6—C8—H8A	109.2	C17'—C18'—H18E	109.5
N2—C8—H8B	109.2	H18D—C18'—H18E	109.5
C6—C8—H8B	109.2	C17'—C18'—H18F	109.5
H8A—C8—H8B	107.9	H18D—C18'—H18F	109.5
O1—C9—N1	125.6 (3)	H18E—C18'—H18F	109.5
O1—C9—N3	126.3 (3)	O7—C19—N3	111.3 (3)
N1—C9—N3	107.9 (3)	O7—C19—H19A	109.4
O2—C10—N2	126.0 (3)	N3—C19—H19A	109.4
O2—C10—N4	125.1 (3)	O7—C19—H19B	109.4
N2—C10—N4	108.9 (3)	N3—C19—H19B	109.4
N2—C11—N1	113.7 (3)	H19A—C19—H19B	108.0
N2—C11—C12	108.6 (3)	O7—C20—N4	111.1 (3)
N1—C11—C12	113.1 (3)	O7—C20—H20A	109.4
N2—C11—C15	102.2 (3)	N4—C20—H20A	109.4
N1—C11—C15	102.3 (3)	O7—C20—H20B	109.4
C12—C11—C15	116.6 (3)	N4—C20—H20B	109.4
O3—C12—O4	125.3 (4)	H20A—C20—H20B	108.0
O3—C12—O4'	127.0 (7)		
			
C6—C1—C2—C3	−1.2 (5)	N3—C9—N1—C7	168.9 (3)
C7—C1—C2—C3	179.5 (3)	N2—C11—N1—C9	94.8 (3)
C1—C2—C3—C4	−1.0 (5)	C12—C11—N1—C9	−140.8 (3)
C1—C2—C3—Br1	177.5 (3)	C15—C11—N1—C9	−14.6 (3)
C2—C3—C4—C5	2.2 (6)	N2—C11—N1—C7	−56.0 (4)
Br1—C3—C4—C5	−176.2 (3)	C12—C11—N1—C7	68.4 (4)
C2—C3—C4—Br2	−178.3 (3)	C15—C11—N1—C7	−165.4 (3)
Br1—C3—C4—Br2	3.2 (5)	C1—C7—N1—C9	−72.5 (4)
C3—C4—C5—C6	−1.1 (6)	C1—C7—N1—C11	76.0 (4)
Br2—C4—C5—C6	179.4 (3)	O2—C10—N2—C11	173.0 (3)
C4—C5—C6—C1	−1.2 (5)	N4—C10—N2—C11	−9.2 (4)
C4—C5—C6—C8	178.6 (3)	O2—C10—N2—C8	6.8 (5)
C2—C1—C6—C5	2.3 (5)	N4—C10—N2—C8	−175.4 (3)
C7—C1—C6—C5	−178.5 (3)	N1—C11—N2—C10	−107.8 (3)
C2—C1—C6—C8	−177.5 (3)	C12—C11—N2—C10	125.4 (3)
C7—C1—C6—C8	1.7 (5)	C15—C11—N2—C10	1.7 (3)
C2—C1—C7—N1	117.2 (4)	N1—C11—N2—C8	58.5 (4)
C6—C1—C7—N1	−62.0 (4)	C12—C11—N2—C8	−68.3 (4)
C5—C6—C8—N2	−120.8 (3)	C15—C11—N2—C8	168.0 (3)
C1—C6—C8—N2	59.0 (4)	C6—C8—N2—C10	86.8 (4)
N2—C11—C12—O3	−28.4 (5)	C6—C8—N2—C11	−78.2 (4)
N1—C11—C12—O3	−155.6 (4)	O1—C9—N3—C19	27.9 (5)
C15—C11—C12—O3	86.2 (5)	N1—C9—N3—C19	−156.0 (3)
N2—C11—C12—O4	142.1 (5)	O1—C9—N3—C15	170.8 (3)
N1—C11—C12—O4	14.9 (6)	N1—C9—N3—C15	−13.1 (4)
C15—C11—C12—O4	−103.3 (5)	O7—C19—N3—C9	93.6 (4)
N2—C11—C12—O4'	169.3 (9)	O7—C19—N3—C15	−47.3 (4)
N1—C11—C12—O4'	42.0 (9)	N4—C15—N3—C9	−107.8 (3)
C15—C11—C12—O4'	−76.1 (9)	C16—C15—N3—C9	127.6 (3)
O3—C12—O4—C13	−10.4 (9)	C11—C15—N3—C9	3.9 (3)
O4'—C12—O4—C13	92.9 (17)	N4—C15—N3—C19	36.5 (4)
C11—C12—O4—C13	179.4 (5)	C16—C15—N3—C19	−88.1 (4)
C12—O4—C13—C14	−173.3 (7)	C11—C15—N3—C19	148.2 (3)
O3—C12—O4'—C13'	10.1 (19)	O2—C10—N4—C15	−169.0 (3)
O4—C12—O4'—C13'	−86 (2)	N2—C10—N4—C15	13.2 (3)
C11—C12—O4'—C13'	171.6 (13)	O2—C10—N4—C20	−32.8 (5)
C12—O4'—C13'—C14'	162 (2)	N2—C10—N4—C20	149.4 (3)
N2—C11—C15—N4	5.9 (3)	N3—C15—N4—C10	99.8 (3)
N1—C11—C15—N4	123.9 (3)	C16—C15—N4—C10	−136.3 (3)
C12—C11—C15—N4	−112.2 (3)	C11—C15—N4—C10	−11.6 (3)
N2—C11—C15—N3	−111.9 (3)	N3—C15—N4—C20	−37.5 (4)
N1—C11—C15—N3	6.1 (3)	C16—C15—N4—C20	86.4 (4)
C12—C11—C15—N3	130.0 (3)	C11—C15—N4—C20	−148.9 (3)
N2—C11—C15—C16	128.0 (3)	O7—C20—N4—C10	−83.8 (4)
N1—C11—C15—C16	−114.0 (3)	O7—C20—N4—C15	49.8 (4)
C12—C11—C15—C16	9.8 (4)	O5—C16—O6—C17	12.8 (8)
N4—C15—C16—O5	117.6 (5)	C15—C16—O6—C17	−163.9 (5)
N3—C15—C16—O5	−117.0 (5)	O5—C16—O6—C17'	−6.2 (13)
C11—C15—C16—O5	−0.5 (6)	C15—C16—O6—C17'	177.0 (11)
N4—C15—C16—O6	−65.6 (4)	C18—C17—O6—C16	−93.3 (16)
N3—C15—C16—O6	59.7 (4)	C18—C17—O6—C17'	−48.3 (18)
C11—C15—C16—O6	176.3 (3)	C18'—C17'—O6—C16	−153.5 (19)
O1—C9—N1—C11	−166.1 (3)	C18'—C17'—O6—C17	62.7 (17)
N3—C9—N1—C11	17.8 (4)	N4—C20—O7—C19	−59.7 (4)
O1—C9—N1—C7	−15.0 (5)	N3—C19—O7—C20	58.3 (4)

### Hydrogen-bond geometry (Å, °)

**Table:**

*D*—H···*A*	*D*—H	H···*A*	*D*···*A*	*D*—H···*A*
C19—H19B···O2^i^	0.97	2.51	3.353 (4)	146
C17—H17A···Br1^ii^	0.97	2.94	3.625 (10)	129
C8—H8B···O2^iii^	0.97	2.39	3.324 (4)	161

Symmetry codes: (i) −*x*, −*y*, −*z*+1; (ii) −*x*, *y*−1/2, −*z*+3/2; (iii) *x*, −*y*+1/2, *z*+1/2.
